# C-Reactive Protein (CRP) and Leptin Receptor in Obesity: Binding of Monomeric CRP to Leptin Receptor

**DOI:** 10.3389/fimmu.2018.01167

**Published:** 2018-05-29

**Authors:** Manu Sudhakar, Santhi Silambanan, Abhinand S. Chandran, Athira A. Prabhakaran, Ramya Ramakrishnan

**Affiliations:** ^1^Department of Biochemistry, Sri Ramachandra Medical College and Research Institute, Chennai, Tamil Nadu, India; ^2^Department of Computational Biology and Bioinformatics, University of Kerala, Thiruvananthapuram, Kerala, India; ^3^Department of Surgery, Sri Ramachandra Medical College and Research Institute, Chennai, Tamil Nadu, India

**Keywords:** obesity, monomeric C-reactive protein, leptin, leptin receptor, protein–protein docking

## Abstract

While leptin deficiency or dysfunction leads to morbid obesity, obese subjects are characterized paradoxically by hyperleptinemia indicating lack of response to leptin. C-reactive protein (CRP) has been suggested to be a key plasma protein that could bind to leptin. To examine whether CRP interferes with leptin action, mediated through its cell surface receptor, docking studies of CRP with the extracellular domain of the leptin receptor were done employing bioinformatics tools. Monomeric CRP docked with better *Z*-rank score and more non-bond interactions than pentameric CRP at the CRH2–FNIII domain proximal to the cell membrane, distinct from the leptin-docking site. Interaction of CRP with leptin receptor was validated by solid phase binding assay and co-immunoprecipitation of CRP and soluble leptin receptor (sOb R) from human plasma. Analysis of the serum levels of leptin, CRP, and sOb R by ELISA showed that CRP levels were significantly elevated (*p* < 0.0001) in non-morbid obese subjects (*n* = 42) compared to lean subjects (*n* = 32) and correlated positively with body mass index (BMI) (*r* = 0.74, *p* < 0.0001) and leptin (*r* = 0.8, *p* < 0.0001); levels of sOb R were significantly low in obese subjects (*p* < 0.001) and showed a negative correlation with BMI (*r* = −0.26, *p* < 0.05) and leptin (*r* = −0.23, *p* < 0.05) indicating a minimal role for sOb R in sequestering leptin.

## Introduction

Chronic low grade inflammation underlies the development and progression of a number of pathological conditions associated with obesity and insulin resistance in human subjects. An increase in plasma levels of inflammatory markers and acute phase proteins such as C-reactive protein (CRP) is observed in subjects with obesity and associated diseases (1). It has been suggested that these proteins are not merely markers or mediators of the inflammatory process but they also affect the action of adipokines, thus having a direct role in the regulation of adiposity. In this context, the potential role of CRP in modulating the action of leptin in obesity is relevant. Human CRP is a 115 kDa pentameric calcium dependent ligand binding plasma protein comprising of five identical polypeptide subunits. The protomers, each containing 206 amino acid residues, associate non-covalently. It binds to a variety of autologous and extrinsic ligands (2). The pentameric form has been reported to dissociate into a more physiologically active and pro-inflammatory monomeric form (mCRP) which can bind to cell surface receptors (3) and has been implicated in the pathogenesis of inflammatory diseases (4).

Leptin is an important adipocyte-derived lipostatic polypeptide hormone which regulates food intake and energy consumption by modulating anorexigenic pathways in the hypothalamus and regulating peripheral tissue metabolism. It is a 16 kDa non-glycosylated protein made up of 146 amino acids arranged in four α-helix and two loop bundle structures (5–7). It acts through a membrane receptor belonging to the interleukin six receptor families which activates the intracellular JAK/STAT signaling pathway (6, 8, 9). The extracellular part of the leptin receptor (Ob R) comprises of an N-terminal domain, a cytokine receptor homology domain 1 (CRH1), an immunoglobulin domain, a second CRH domain (CRH2), and two FN type III domains proximal to the plasma membrane (10, 11). Leptin binds with high affinity to the CRH2 domain while the proximal FN type III domains seem to be critical in Ob R activation (8, 12). Of the different isoforms of Ob R, the soluble form present in the plasma is reported to be formed by constitutive shedding of the extracellular domain of the membrane-anchored Ob R (13).

Plasma levels of leptin are altered in several pathological conditions, including cardiovascular diseases (14–17), obesity (7, 18), and reproductive disorders (19). Congenital leptin deficiency leads to uncontrolled appetite and morbid obesity in humans (6). However, a majority of obese subjects are characterized, paradoxically, by elevated levels of leptin in plasma suggesting that leptin fails to exert its effect in spite of being present in abundance (20, 21). This state of hyperleptinemia with a reduced response to circulating hormone is independently associated with insulin resistance and coronary heart disease in human subjects (22). Investigations into the molecular mechanism of leptin resistance, carried out mainly in experimental animals and cell-based systems (20, 23, 24), suggested that binding of plasma leptin to circulating molecules such as soluble leptin receptor (sOb R) and acute phase proteins could be an important mechanism that limits the availability and action of leptin at target tissue.

Several clinical studies have shown a correlation between increased plasma levels of both CRP and leptin in subjects with obesity, CVD, and diabetes (25–30). Although a concomitant increase in CRP and plasma leptin has been observed in obesity, it is not clear whether elevation in CRP is due to acute inflammation or due to adipose tissue expansion or both. While leptin is produced by adipocytes (31), CRP is produced primarily by liver and vascular cells (32, 33). Changes in leptin levels were also found to be independently associated with CRP (after adjustment for age, gender, smoking, alcohol consumption) (34) indicating a relationship between leptin and CRP production. This has been further confirmed by demonstrating a direct effect of leptin on CRP production by hepatocytes and suggests that circulating leptin and CRP levels are linked by a regulatory loop (35). This seems to indicate the existence of an adipo–hepato axis whereby leptin produced by adipocytes enhances CRP expression which in turn may antagonize leptin action by limiting its tissue availability (24).

As CRP is a circulating factor which can bind to leptin, its interaction with leptin has been suggested to be critical in regulating the availability of leptin in the hypothalamus and a factor that might contribute to leptin resistance (36). However, no direct proof for a role of CRP in regulating leptin action inside the CNS is available. CRP has also been shown to attenuate leptin signaling in cells overexpressing Ob R (36), however it was not clear whether this was a result of binding with leptin or a direct effect of CRP on Ob R. Therefore, in the present study we have examined the possible interaction of CRP with the leptin receptor and its implication in obesity. Results of the study showed that CRP, particularly the monomeric CRP, binds to sOb R, whose level in the plasma is decreased in obese subjects.

## Materials and Methods

### Materials

All the chemicals used were high quality analytical grade reagents procured from Merck, Mumbai, India, Spectrochem, Mumbai and SRL Mumbai, India. Plastic wares used were products of Becton & Dickinson, Tarson India and NUNC. ELISA kits for assay of leptin and leptin receptor were procured from Invitrogen USA. Human CRP and recombinant leptin receptor (synthesized in *Escherichia coli*) were procured from Thermo Fischer, USA. Purified antibody against leptin receptor (rabbit IgG, HPA030899), human CRP (mouse IgG, C1688), HRP conjugated anti-rabbit IgG, HRP conjugated anti-mouse IgG, protein A-sepharose and o-phenylamine diamine (OPD), and DAB were the products of Sigma Aldrich, USA. NC membrane and Clarity Western ECL substrate were products of Bio Rad, USA.

### Methods

#### Protein–Protein Docking Studies

Interaction of leptin, CRP, and Ob R was studied by protein–protein docking tools using Discovery Studio 4.0. The crystal structures of CRP (PDB ID. 1GNH), human leptin (PDB ID. 1AX8), and Ob R (PDB ID.3v6O) were taken from PDB database. The Z dock protocol (37) in Discovery studio 4.00 (DS) was used to perform docking of proteins. The protein structures downloaded from the PDB database were prepared for docking using “Prepare protein protocol.” The monomer structure was taken from the prepared pentameric structure of CRP. The missing amino acid residues 24–39 in the leptin structure were inserted using optimize side chain conformation for residues with inserted atoms. Leptin receptor was modeled as described below and optimized for docking. Z Dock algorithm in DS used the Fast Fourier Transform correlation technique to search all possible binding positions of the docking proteins; it performed an exhaustive multi-dimensional search in the translational and rotational space between two docking molecules. The scoring function of Z Dock is a geometrical measure according to the degree of shape complementarity between the docking proteins. The Z Dock prediction was re-ranked to yield *Z*-rank score which is a linear weighted sum of van der Waal’s energy, electrostatic attractive and repulsive energies, and degree of solvation. The Z-Dock score is expressed as positive values and the *Z*-rank score is expressed as negative values. An evenly distributed rotational search gave 2,000 poses of which the best ranked pose represented by the lowest *Z*-rank score (38, 39) in the largest cluster was taken for comparison and the analysis of the docking interactions.

##### Homology Modeling of Leptin Receptor

Homology modeling of leptin receptor was done using the crystal structure of CRH2 domain of Ob R (PDB ID 3V6O) and the FnIII domain of gp130 (PDB ID.3L5I_A) as templates. Using psi BLAST program, sequence similarity search was done against protein sequences in PDB depository and the templates for residues 431–841 in the CRH2 and Fn III domains of the extracellular domain of leptin receptor were identified. 3D model was built using MODELER program in DS and compared the model structure with the template to evaluate the model score. The verified score value of the modeled receptor (92.2499) was higher than the expected low score value (84.3719).

#### Binding Assay

For binding studies, monomeric CRP (mCRP) was prepared from human serum CRP or recombinant CRP by treatment with 8 M urea/10 mM EDTA at 37°C for 2 h as described in Ref. (40). Binding of leptin receptor to CRP was assayed following an ELISA method similar to that described before (3). 96-well microtiter plate was coated with different concentrations of mCRP by incubating at 4°C overnight in Tris/HCl buffer (0.05 M, pH 7.4). Free binding sites were blocked by incubating with 0.5% gelatin in PBS (0.15 M NaCl, 0.005 M phosphate buffer, pH 7.2, 0.05% Tween20) at room temperature for 2 h. Soluble recombinant human leptin receptor (100 ng/ml) in 50 µl PBS was added to the wells and incubated at RT for 1 h. Wells without CRP coating served as control. Non bound Ob R was removed, washed with PBS as above, and each well was treated with 50 µl anti-Ob R antibody (1:1,000) for 1 h; the antibody was removed at the end of the incubation, the wells washed thrice with PBS and treated with HRP conjugated anti-rabbit IgG (1:1,000) for 1 h. At the end of the incubation, HRP was removed; the wells washed thrice with PBS, and developed using OPD/H_2_O_2_ as substrate. The bound Ob R was quantified by measuring the absorbance at 480 nm.

##### Co-Immunoprecipitation of sOb R and CRP From Serum

To 50 μl freshly isolated human serum pre-treated with protein A sepharose, 5 µl of 10× PBST (1.5 M NaCl, 0.05 M phosphate buffer, pH 7.2, 0.5% Tween 20) and 0.5 µl of anti-Ob R or anti-CRP were added and incubated overnight at 4°C followed by incubation at room temperature with protein A sepharose for 2 h. The protein A beads were collected by centrifugation at 500 *g* for 10 min and washed three times with PBST and finally with PBS. The beads were then treated with 50 µl of 0.1% SDS in PBS, centrifuged and the co-precipitated CRP or sOb R determined in an aliquot by ELISA with anti-CRP or anti-Ob R antibody (1:1,000) using HRP-conjugated anti-mouse IgG or anti-rabbit IgG as described above. Serum samples treated with protein A sepharose without adding antibody were taken as control. The specificity of the anti-Ob R antibody tested by Western blotting using human serum showed the presence of major and a minor band in molecular size 120–150 kDa similar to that reported earlier (41).

For Western blot analysis, serum samples pre-treated with protein A beads were immunoprecipitated with anti-Ob R antibody as above, the immuno-precipitate captured with protein A beads, the beads washed with PBST, extracted with electrophoresis sample buffer, subjected to SDS-PAGE (42), and electro-blotted onto nitrocellulose membrane (43) using a Biometra Transblot apparatus. Blotted protein was probed with anti-CRP antibody (1:1,000 in PBST) followed by HRP conjugated second antibody (1:1,000 in PBST/0.5% BSA) and developed using ECL reagent. A negative control without addition of the primary antibody while probing was also carried out.

#### Analysis of CRP, Leptin, and sOb R in Serum—Study Design and Subjects

A total of 79 subjects, 50 males and 29 females, attending the master health check-up at Sri Ramachandra medical college hospital, Chennai were part of this sub-group study. Subjects were aged between 22 and 45 years. There were 32 lean subjects (21 males and 11 females), 5 overweight subjects, and 42 obese subjects (28 males and 14 females). Subjects were considered lean if their body mass index (BMI) was less than 23 kg/m^2^, overweight if it was between 23 and 25 kg/m^2^, and obese if it was greater than 25 kg/m^2^ (according to World Health Organization, Western Pacific region guidelines). Those with a known history of diabetes mellitus, hypertension/systolic blood pressure of >140 mm Hg or diastolic blood pressure >90 mm Hg at the time of examination, coronary artery disease, cerebrovascular accident, bronchial asthma, cancer as well as those on lipid lowering therapy, anti-thrombotic therapy, steroids, those who had surgery within the previous 3 months, pregnant women, and lactating mothers were excluded. The study was approved by the Institutional Ethics Committee of the Sri Ramachandra University (Ref. IEC-NI/12/DEC/31/62) and all subjects provided informed written consent. The study was carried out in accordance with the ethical standards of the Institutional Ethics Committee and with the 1964 Helsinki declaration and its later amendments or comparable ethical standards.

##### Anthropometric Measurements

The height, weight, waist circumference, and the hip circumference were measured and BMI calculated as weight (kg)/square of height (m). The blood pressure was measured in the left brachial artery in the sitting position by auscultatory method using a sphygmomanometer.

##### Biochemical Analyses

Blood samples were collected from subjects, after an overnight fast, in yellow topped BD vacutainers with a separator gel, allowed to stand for 30 min, centrifuged at 5,000 rpm for 10 min, and the serum separated into two aliquots. The first was immediately analyzed for lipids and CRP, while the other was stored at −40°C and after completion of sample collection, analyzed for leptin and sOb R. Another blood sample was collected in a sodium fluoride containing gray topped BD vacutainers and centrifuged immediately to obtain plasma for analysis of fasting plasma glucose. Plasma glucose, total cholesterol, HDLc, LDLc, and triglycerides were measured by the enzymatic photometric methods on the Siemens Advia 1800 auto analyzer using commercial kits following manufacturer’s recommendations. Serum CRP was measured by particle enhanced turbidimetric immunoassay using the Dade Behring RXL Max autoanalyzer. Concentration of leptin in serum was determined using the Invitrogen Hu Leptin ELISA kit (the sensitivity of the assay was 3.5 pg/ml; intra- and inter-assay coefficient of variations for lowest and highest values were 3.0 and 3.8% and 3.9 and 4.6%, respectively) while the levels of sOb R were assayed by Quantikine sOb R ELISA kit of R&D systems (the sensitivity of the assay was 0.057 ng/ml; intra- and inter-assay coefficient of variations for lowest and highest values were 6.1 and 4.9% and 8.6 and 6.8%, respectively).

### Statistical Analysis

All serum values are expressed as mean ± SEM or median (IQR). After data were tested for normality by the Shapiro–Wilk test, comparison was done either by the unpaired “*t*” test with Welch’s correction or Mann–Whitney *U* test. Correlation analysis was done using the Spearman coefficient of correlation. A “*p*” value of less than 0.05 was considered significant. Statistical analysis was done using Graph Pad Prism 5.01.

## Results

### Interaction of CRP With Leptin Receptor; Docking of CRP to Leptin Receptor

**Figure 1:**
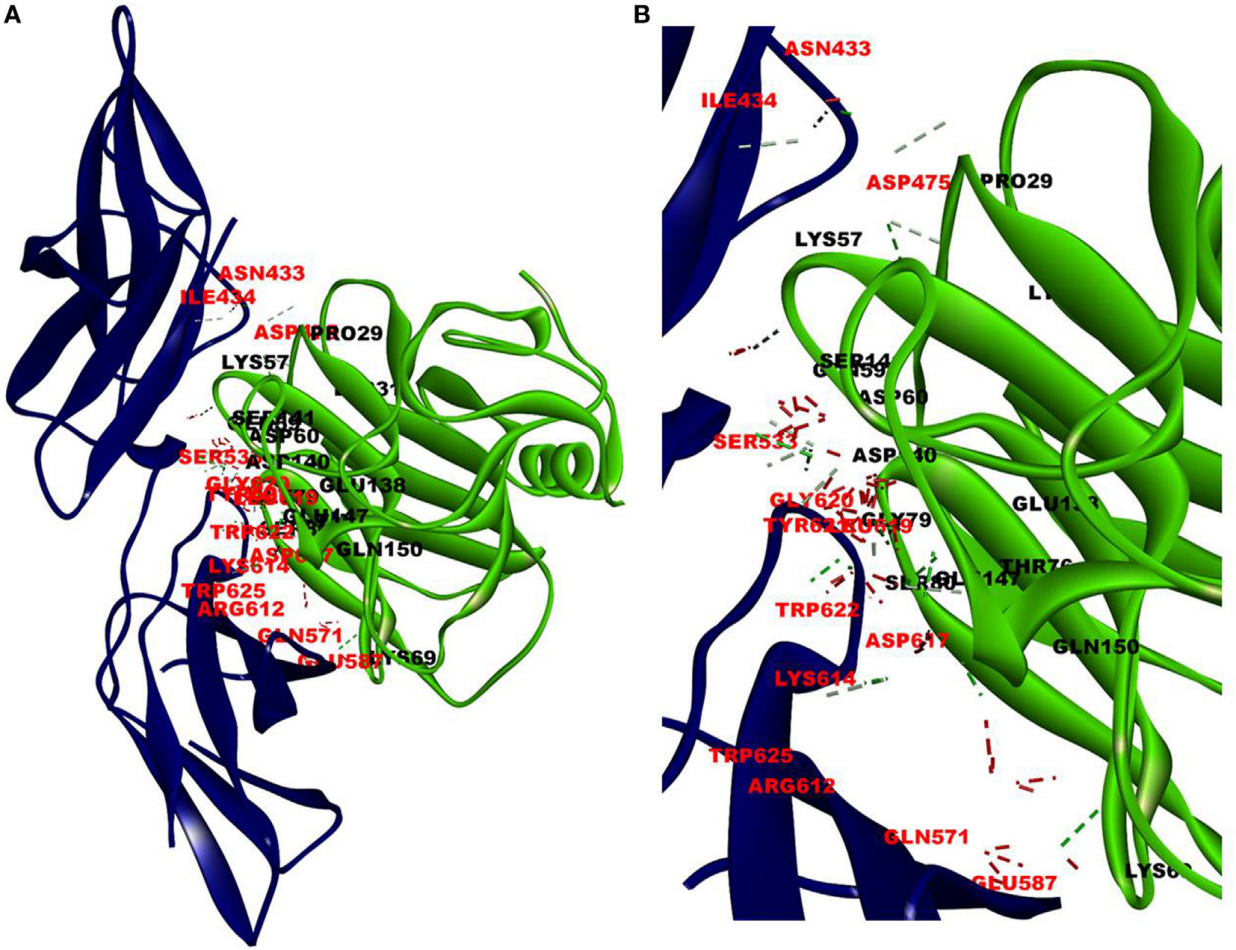
Docking of C-reactive protein (CRP) to leptin receptor. Protein–protein docking was done by employing Z Dock Protocol in Discovery Studio as described in the text. The image of the pose with the best *Z*-rank score in the largest cluster is shown in **(A)** leptin receptor, PDB ID 3V6O, (CRH2 domain), (deep blue) docked (blind) with CRP monomer, PDB Id 1GNH, (single chain) (green) and **(B)** enlarged image of docked amino acid skeleton.

**Figure 2:**
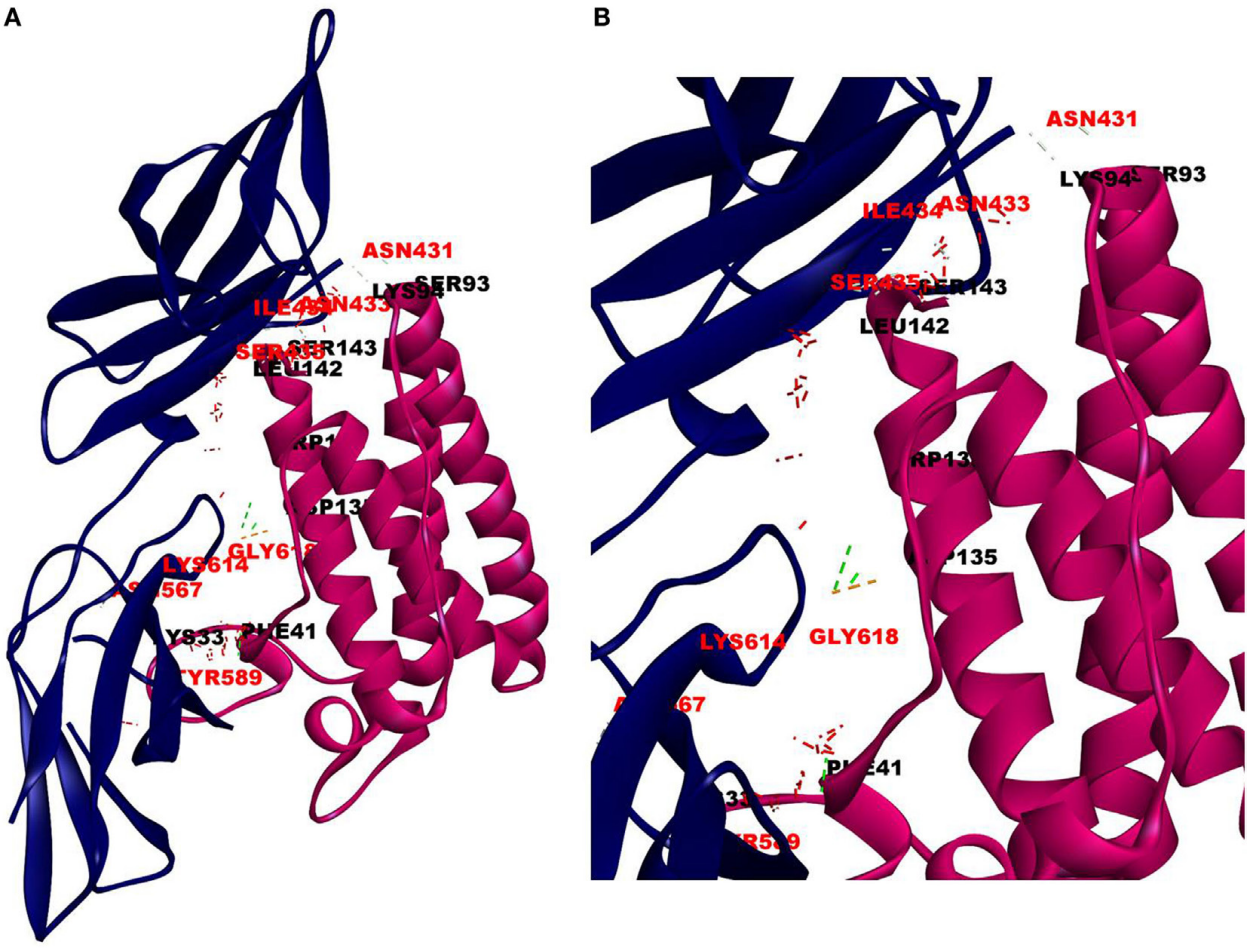
Docking of leptin to leptin receptor. Protein–protein docking was done between leptin and leptin receptor (CRH2, PDB ID 3V6O) as described in legends to Figure 1. The image of the pose with the best *Z*-rank score in the largest cluster is shown in **(A)** leptin, PDBID-1AX8, (pink) docked (blind) with leptin receptor, PDB ID 3V6O, (CRH2 domain) (deep blue) and **(B)** enlarged image of docked amino acid skeleton.

The cellular effect of leptin involves its binding to the extracellular domain of the cell surface receptor followed by downstream signaling. To examine whether CRP interacts with the leptin receptor, docking studies of CRP to the extracellular domain of the receptor was done. For this, initially, the CRH2 domain of the receptor that has been shown to be sufficient to induce leptin response (8) was employed in the docking with mCRP and the results are shown in Figure 1. The best ranked pose in the largest cluster (*Z*-rank score −115.571) was found to involve 22H-bond interactions between the two docking partners (Table S1A in Supplementary Material); mCRP interacted with the amino acid residues 619(L), 620(G), 621(Y), 622(W), and 625(W), mostly present in the C-terminal region of CRH2 domain of the Ob R. To examine whether the mCRP docking site is same or different from that of leptin-binding site on CRH2, docking of leptin to the CRH2 domain was done (Figure 2). The best pose in the largest cluster, (*Z*-rank score of −113.578), showed leptin interacting through 12H-bond interactions predominantly with residues 431N, 433N, 434I, 435S, 567N, 589Y, and 614K of the CRH2 domain (Table S1B in Supplementary Material) indicating that leptin and CRP dock at different sites on the leptin receptor. However, similar docking studies using pentameric CRP with leptin receptor showed that the best pose gave a poor *Z*-rank score and less number of non-bond interactions than those shown by mCRP (Table S1C in Supplementary Material). These results suggest that leptin mostly docks to the residues toward the N-terminal region of the CRH2 domain, while mCRP docks to residues toward its C-terminal region closer to the plasma membrane.

**Figure 3:**
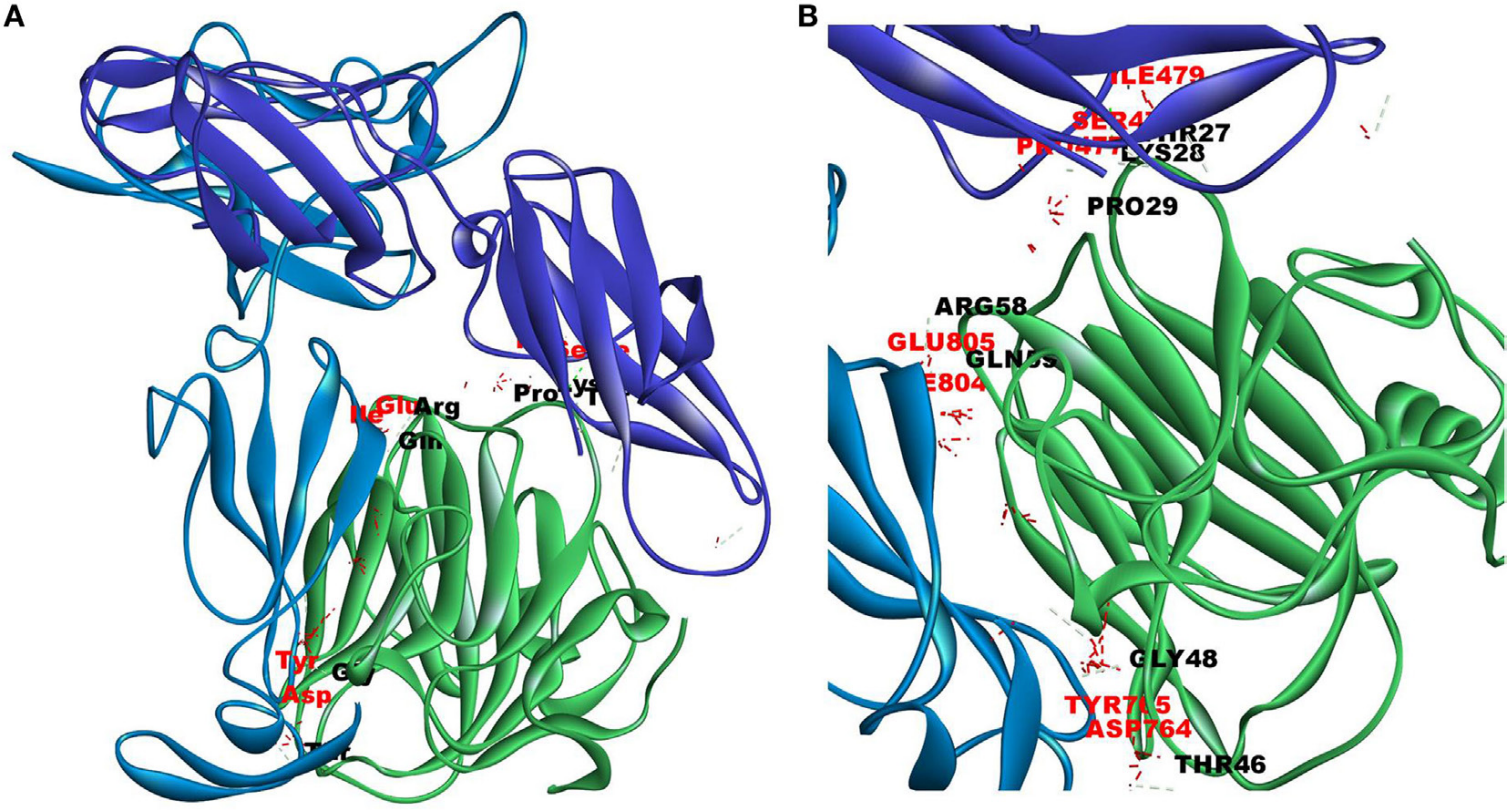
Docking of C-reactive protein (CRP) to modeled leptin receptor. Protein–protein docking was done between modeled leptin receptor (CRH2–FNIII) and CRP (monomer) (PDB Id 1GNH) as described in Figure 1. The image of the pose with the best *Z*-rank score in the largest cluster is shown in **(A)** leptin receptor modeled [CRH2 (deep blue) –Fn III domains (light blue)] docked with CRP monomer (green) and **(B)** enlarged image of docked amino acid skeleton.

**Figure 4:**
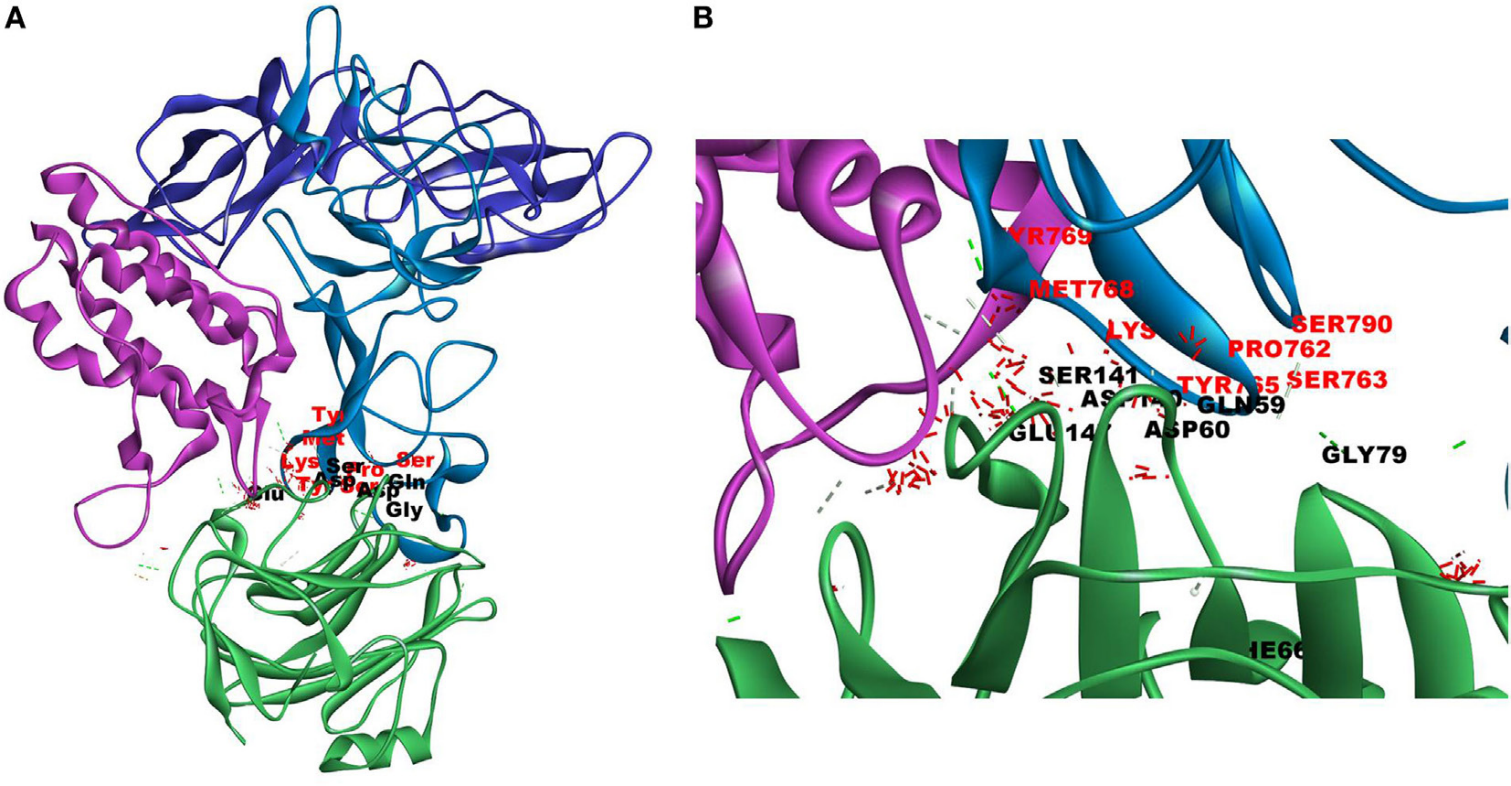
Docking of C-reactive protein (CRP) to leptin–leptin receptor complex. Leptin (PDB Id 1AX8) –leptin receptor (CRH2–FNIII) complex was prepared, the complex was energy minimized, prepared for further docking as described in Section “Materials and Methods” and docked with CRP (PDB Id 1GNH). Protein–protein docking was done as described in Figure 1. The image of the pose with the best *Z*-rank score in the largest cluster is shown. **(A)** Leptin (pink) was docked (site specific) to modeled leptin receptor [CRH2 (deep blue)–Fn III (light blue)], CRP monomer (green). **(B)** Enlarged image of docked amino acid skeleton.

Since mCRP was found to dock to C-terminal region of the CRH2 domain which is linked to the FN type III domain of the Ob R and is reported to be essential for Ob R activation and signaling (8), the possible role of this domain if any in the interaction of mCRP was examined. The Ob R (CRH2–FNIII) was modeled and prepared for docking as described above. mCRP docked to the FNIII domain (*Z*-rank score of −121.633) with 13H-bond interactions with the amino acid residues (764)NYKL(767), 794K, (803)PIE(805), 807Y in FN type III domain and 474D, (477)PSIH(480), 517H, 518S, 522l, 523D, 525P, and 526P in the CRH2 domain (Figure 3) (Table S1D in Supplementary Material). These results indicate that mCRP can interact with the membrane proximal FN type III domain and CRH2 domain, unlike leptin which docked to the membrane distal region of the CRH2 domain of Ob R. In the light of the earlier report on binding of CRP to leptin (36), it was proposed to examine whether CRP docks to leptin or its receptor in the leptin–receptor complex. For this, a leptin-Ob R structure was created *via* Z dock, using the Ob R containing the CRH2–FN type III domain model described above. The best ranked pose was energy minimized using standard dynamics cascade and the prepared proteins were further processed for docking with mCRP. The best ranked pose had a *Z*-rank score of −129.234 and involved 17H-bond interactions. This showed that CRP docked predominantly to the receptor component (762P, 763S, 765Y, 766K, 768M, 769Y, 790S, 829T, 830Q, and 832D) (Table S1E in Supplementary Material) on the FN III domain. However, certain residues on leptin (35K, 117S, 118G, and 119Y), which are not involved in docking to the Ob R, dock with CRP in the leptin–receptor complex (Figure 4).

A comparison of the *Z*-rank scores which is indicative of the energy, and thus of the stability of the structures, showed that mCRP docked best with the Ob R having CRH2–FN III domain (Table 1) and the amino acid residues predominantly in the membrane proximal domain of the leptin receptor were involved in docking. The Z-dock score, which is a geometrical measure of the degree of shape complementarity and thus a reflection of the binding ability was better for docking of CRP to Ob R than that of leptin to Ob R.

**Table 1:** Docking scores and H-bonding interactions of CRP and LR.

Sl No	Molecules docked	*Z*-rank score	Non bond interactions (<3Å)
1	L–LR (CRH2)	−113.578	12H
2	LR (CRH2)–CRP monomer	−115.571	22H
3	LR (CRH2)–CRP pentamer	−109.101	8H
4	LR (CRH2, Fn)–CRP monomer	−121.633	8H
5	L LR(CRH2, Fn)–CRP	−129.234	16H

*L, leptin; LR, leptin receptor; CRP, C-reactive protein; LR(CRH2), cytokine receptor homology domain 2 of LR, LR(CRH2-Fn), CRH2-fibronectin type III domains of LR. Residues within a distance of 3Å involved in H-bond interactions were considered*.

### Binding of mCRP to Leptin Receptor

**Figure 5:**
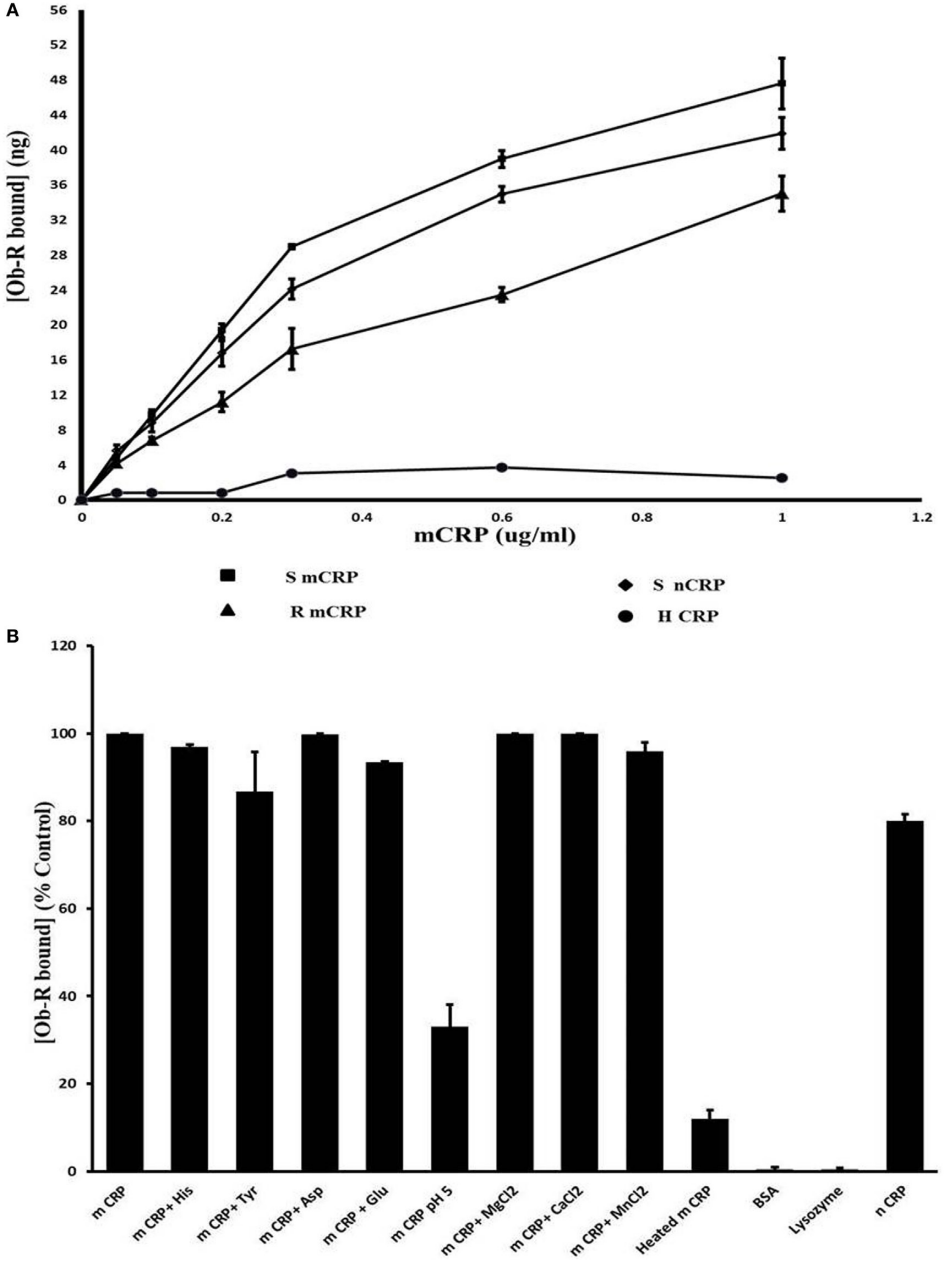
Solid phase binding assay of leptin receptor to C-reactive protein (CRP). **(A)** Concentration dependence: different concentrations of monomeric CRP (mCRP) from serum (s-mCRP), recombinant CRP (R-mCRP), native serum CRP (n-CRP), and heat treated (h-mCRP) were coated on to 96-well micro titer plate incubated with leptin receptor (100 ng of Ob R) and the amount of Ob R bound was quantitated by ELISA using anti-Ob R antibody and HRP-conjugated secondary antibody as described in text. Values given are the average of 3–6 experiments done in duplicate ± SD. **(B)** Effect of different substances: mCRP (0.6 µg/ml) was coated on to 96-well micro titer plate and binding of leptin receptor in the presence of different cations (1 µM each of MnCl_2_, MgCl_2_, and CaCl_2_), or different amino acids (1 µM each of His, Tyr, Asp, and Glu) or in buffer pH 5.0 was assayed. Binding of leptin receptor to wells coated with BSA (100 µg/ml), lysozyme (100 µg/ml) native CRP (nCRP), or heat-treated CRP (0.6 µg/ml) was also analyzed. Results are expressed as percent of untreated mCRP control. Each value represents mean ± SD of three experiments.

Results of docking studies were tested using an ELISA type solid phase binding assay and the results are shown in Figure 5. Leptin receptor bound to immobilized mCRP in a concentration-dependent manner (Figure 5A). mCRP prepared from human serum CRP showed better binding than recombinant mCRP. The K_d_ for binding to human mCRP was 1.02 × 10^−8^ M. Heat-treated mCRP did not show any binding to the leptin receptor indicating that the native structure of CRP is critical in binding to the leptin receptor. Native pentameric CRP was also found to bind to the leptin receptor, though to a lesser extent than mCRP (80%). Presence of Ca^2+^, Mg ^2+^, or Mn^2+^ ions did not affect the binding. The binding appeared to be specific as non-relevant proteins such as serum albumin or lysozyme did not show significant binding (Figure 5B). However, amino acids, such as His, Tyr, Asp, and Gln, which were predicted to be involved from docking studies, did not cause any significant effect at the concentrations tested. However, a decrease in pH 5 in the binding assay medium caused a significant reduction in the binding, indicating that protonation of certain residues might have affected the binding.

### Co-Immunoprecipitation of sOb R and CRP of Serum

**Figure 6:**
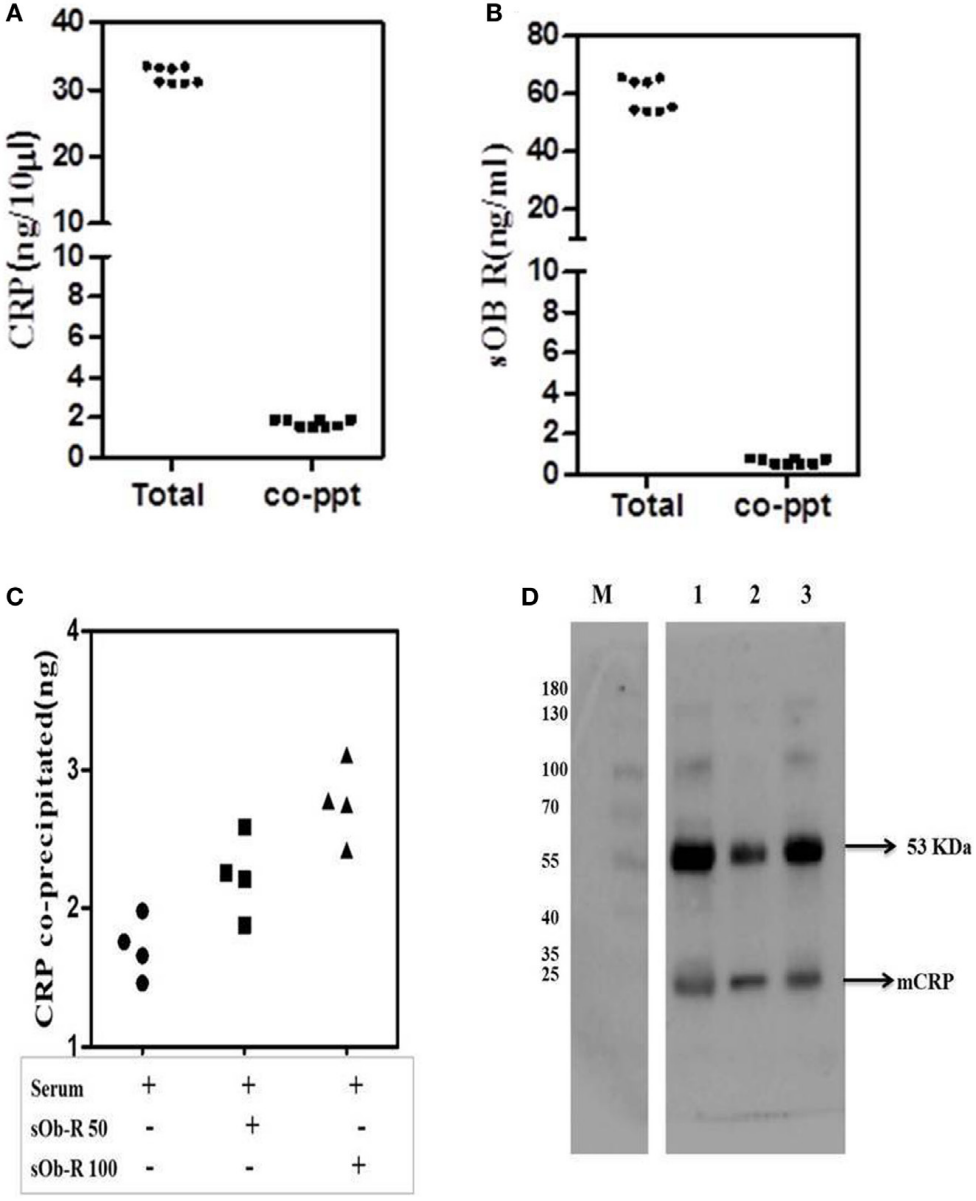
Co-precipitation of soluble leptin receptor (sOb R) and C-reactive protein (CRP) from serum. **(A)** Co-precipitation of CRP with sOb R: sOb R was immunoprecipitated from fresh human serum and the amount of CRP present in the immunoprecipitate was assayed by ELISA. Total CRP in serum was also assayed. Scatter plot represents results of multiple experiments in replicates. (mean ± SD of total CRP was 32.18 ± 1.24 and that of co-precipitated CRP was 1.74 ± 0.17).**(B)** Co-precipitation of soluble leptin receptor with CRP: CRP was immunoprecipitated from freshly isolated human serum and the amount of soluble leptin receptor present in the immunoprecipitate was determined by ELISA. Total soluble leptin receptor in serum was also determined. [mean ± SD of total soluble leptin receptor (sOb R) was 59.6 ± 5.6 and that of co-precipitated sOb R was 0.67 ± 0.14]. **(C)** Co-precipitation of CRP from serum spiked with different concentrations of soluble leptin receptor. To 50 µl of freshly isolated serum, different concentrations (50, 100 ng) of leptin receptor were added, incubated for 30 min, immunoprecipitated leptin receptor, and estimated the amount of CRP co-precipitated by ELISA as above. Scatter plot represents results of multiple assays and shows an increase in co-precipitated CRP with increase in concentrations of added leptin receptor. **(D)** Immunoblot analysis of co-precipitated CRP. sOb R was immunoprecipitated from human serum, subjected to 7.5% SDS-PAGE followed by Western blotting, probed with anti-CRP, and located by ECL as described in the text. Lane 1: sOb R co-precipitate. Lane 2: immune-precipitated CRP from sOb R co-precipitate. Lane 3: immunoprecipitated CRP from whole serum. M: molecular weight markers.

To further examine binding of CRP to Ob R, sOb R was immunoprecipitated from human serum and the amount of co-precipitated CRP estimated; the results are shown in Figure 6. About 5% of serum CRP was co-preciptated with sOb R. Spiking with different concentrations of sOb R followed by immunoprecipitation showed an increase in the amount of CRP co-precipitated from serum with increase in the amount of added sOb R. Similar analysis of immunoprecipitated CRP showed that about 1.25% of the total sOb R was co-precipitated with CRP. Co-immunopreciptation of CRP with sOb R was confirmed by SDS -PAGE and Western blot analysis which showed the presence of a 24 kDa band corresponding to the monomeric subunit of plasma CRP (Figure 6D lane 1) in the immunoprecipitated sOb R. To further confirm that CRP was co-precipitated, the immuno-precipitate captured on protein-A beads was extracted with 0.1% SDS in PBST, the CRP immunoprecipitated and subjected to Western blotting. Presence of an identical band (Figure 6D lane 2) corresponding to molecular size 24 kDa confirmed the presence of CRP. An identical band was also obtained when whole serum was subjected to similar analysis using anti-CRP antibody (Figure 6D lane 3). Binding of CRP to sOb R was further confirmed by co-immunoprecipitation of sOb R with CRP. CRP was immunoprecipitated from serum by anti-CRP and the immunoprecipitate subjected to electrophoresis and immunoblotting using antibody against sOb R. Two bands in the molecular weight region 121 and 136 kDA, corresponding to sOb R, were found suggesting co-precipitation of sOb R with CRP (Figure S1 in Supplementary Material).

In both instances, another band corresponding to an approximate molecular weight 53 kDa was observed. This appeared to be the immunoglobulin detected by the secondary antibody as suggested by its persistence in negative controls which did not use a primary antibody for probing. The antigens of interest, CRP and sOb R were not detected in negative controls confirming specificity of the primary antibody (Figure S1 in Supplementary Material).

### Serum Levels of leptin, CRP, and sOb R in Lean and Obese Human Subjects

As an important protein in plasma that binds to leptin, the level of sOb R in plasma is critical in determining the availability of leptin and its response. Earlier studies in human subjects with obesity associated metabolic diseases obtained contrasting results with either a increase, no change, or a decrease in plasma levels of sOb R (44). It is not clear whether these variations are due to associated metabolic complications of obesity. To assess the relation, if any, between the concentration of sOb R and its binding proteins, particularly CRP and leptin, the concentrations of these proteins in serum of lean and obese human subjects without any history of co-morbidities, as described in Section “Materials and Methods,” was determined. The anthropometric and biochemical profile of the subjects are given in the Table S2 in Supplementary Material. None of the subjects had any history of acute or chronic inflammatory disorders or infection. There was no significant difference between the age of obese and lean subjects. While there was no significant difference between levels of total cholesterol of the two groups, there was a significant decrease in HDLc and an increase in LDLc levels and triglyceride levels in obese subjects. Although the FBS levels were higher in obese subjects, none of the subjects were diabetic.

**Figure 7:**
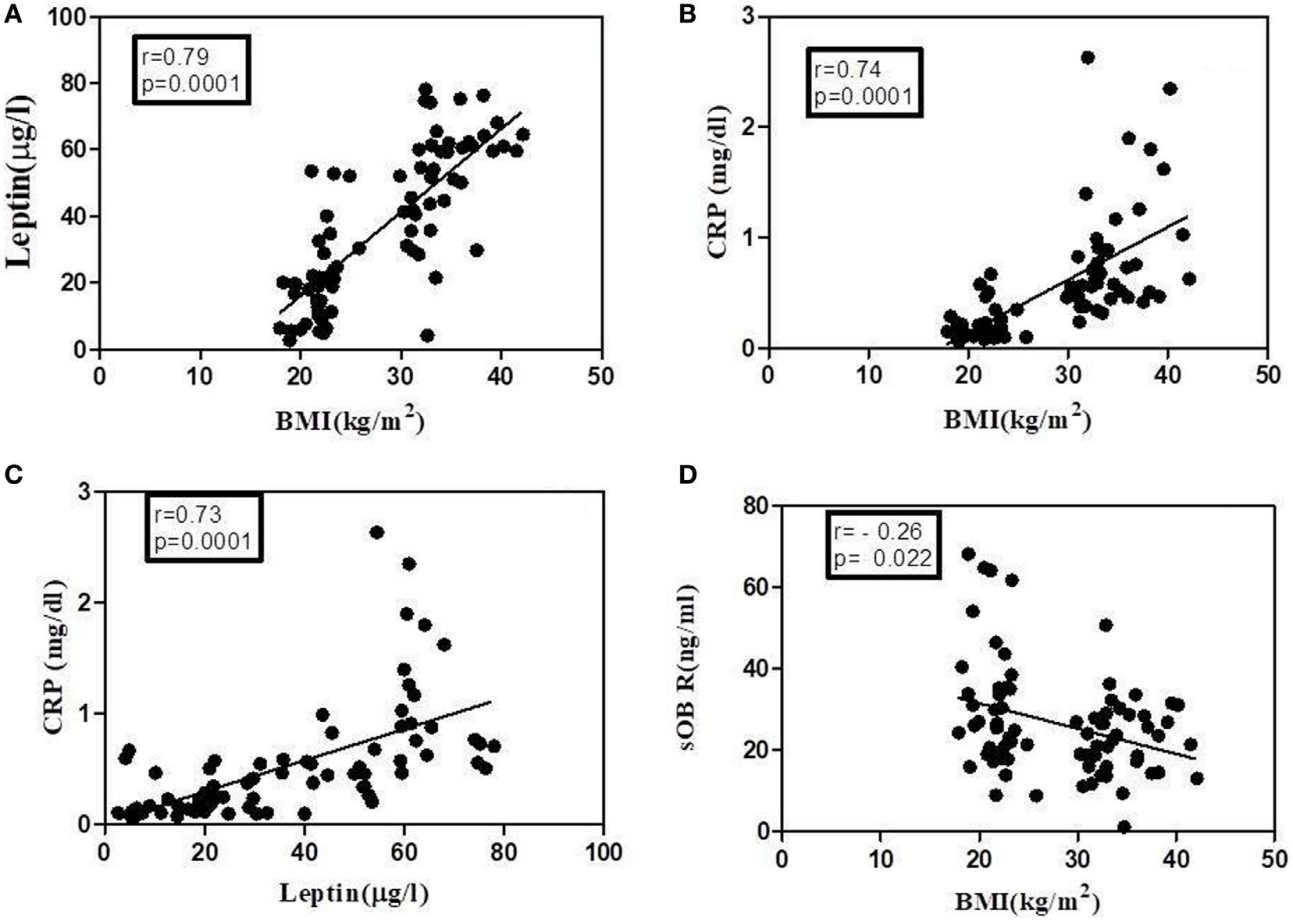
Correlation of leptin, C-reactive protein (CRP), and soluble leptin receptor (sOb R) with body mass index (BMI) analyses of correlation of BMI with serum levels of leptin, CRP, and sOb R as well as that between leptin and CRP was done using Spearman coefficient of correlation. A value of *p* < 0.05 was considered significant. Dark circles represent individual values. **(A)** BMI and leptin (*n* = 78, *r* = 0.79), **(B)** BMI and CRP (*n* = 78, *r* = 0.74), **(C)** leptin and CRP (*n* = 77, *r* = 0.73), and **(D)** BMI and sOb R (*n* = 79, *r* = −0.26).

The levels of leptin, sOb R, and CRP in the serum of these subjects are given in Table 2. A significant increase in the levels of leptin in serum of obese subjects, compared to lean subjects, was observed, consistent with the earlier reports (45). There was a positive correlation between levels of leptin and BMI (Figure 7A). In agreement with earlier reports, serum levels of CRP showed a significant increase in obese subjects when compared to that of lean subjects. The levels of CRP correlated positively with both BMI and serum leptin levels (Figures 7B,C). The levels of sOb R in the serum of obese subjects was significantly lower than that in lean subjects; it showed a negative correlation with BMI (Figure 7D) and serum levels of leptin (*r* = −0.23, *p* < 0.05). Though there was decrease in sOb R, no significant negative correlation between CRP and sOb R levels was observed. Increase in levels of serum CRP and leptin were observed in both male and female obese subjects (Table S3 in Supplementary Material). However, no significant difference in sOb R was found between lean and obese female subjects.

**Table 2:** Serum levels of leptin, soluble leptin receptor (sOb R), and C-reactive protein (CRP) in lean and obese subjects.

	Body mass index (BMI) <23	BMI >25	*p*
*N*	32	42	
Leptin (ng/ml)	18.0 (7.6–21.5)	54.6 (40.95–62.2)	<0.0001
sOb R (ng/ml)	26.38 (19.8–35.36)	21.37 (15.65–28.04)	<0.05
CRP (mg/dl)	0.16 (0.11–0.23)	0.60 (0.465–0.95)	<0.0001

*Values expressed as median (interquartile range). Comparison was done by the Mann–Whitney *U*-test*.

**Figure 8:**
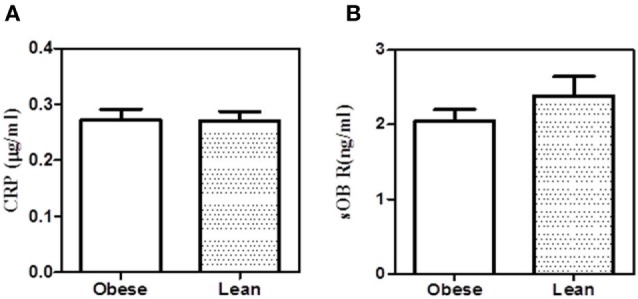
Co-precipitation of leptin receptor and C-reactive protein (CRP) from serum of obese and lean subjects. **(A)** Soluble leptin receptor (sOb R) was immunoprecipitated from the serum of lean (*n* = 6) (body mass index, BMI <23) and obese (*n* = 5) (BMI >25) male subjects and CRP estimated by ELISA as shown in Figure 3. **(B)** CRP was immunoprecipitated from the serum of lean and obese male subjects and estimated sOb R by ELISA. Values given are the mean ± SD. *p* > 0.05 not significant.

To examine whether the binding of sOb R to CRP in plasma was affected in obese condition, the levels of sOb R and CRP co-immunoprecipitated were analyzed as done above. The amount of CRP co-precipitated with sOb R from serum was not significantly different in obese subjects compared to lean subjects (Figure 8). The amount of sOb R co-precipitated with CRP was also not different.

## Discussion

Investigations into the molecular mechanisms of leptin resistance have led to the suggestion that circulating leptin-binding proteins could limit its availability at target sites. Among the different proteins in the plasma, CRP, a major acute phase protein has been considered as the principal leptin-binding protein that could limit the action of leptin. Results presented above suggest that CRP does interact with the leptin receptor. The evidence in support of binding of CRP with the extracellular domain of the Ob R includes (a) molecular docking of CRP to extracellular CRH2–FN type III domain of Ob R (b) binding of the recombinant leptin receptor to CRP, particularly to mCRP, in a solid phase binding assay, and (c) co-immunoprecipitation of CRP with sOb R from human serum.

Molecular docking studies revealed that mCRP docks to the extracellular domain of the leptin receptor. Detailed analysis of the docking interactions revealed the following: (a) mCRP, but not the larger pentameric CRP, docks to the C-terminal region of the CRH2–FNIII domain of Ob R proximal to the plasma membrane. (b) mCRP docking site is distinct from the leptin-docking site which is located more toward the N-terminal region of the CRH2 domain of Ob R. (c) Docking studies with leptin–leptin receptor complex predict that mCRP docks directly to CRH2FNIII domain of the receptor and not through leptin; it docks predominantly to the receptor component with amino acids predominantly in the FNIII domain with minimal interaction with the leptin molecule. (d) A better *Z*-rank score (Table 1), which is a linear weighted sum of the van der Waal’s energy, electrostatic energy, and degree of solvation reflecting the binding energy, predicted that the mCRP–Ob R interaction is ranked comparably with that of leptin, the natural ligand. Comparison of the *Z*-rank scores for docking of mCRP with Ob R (CRH2–FNIII) and leptin–Ob R complex (−121.633 and −129.234) further suggests that leptin may not affect interaction of mCRP with the Ob R. However, rigid docking studies such as these offer limited insights into ligand-induced changes in the binding characteristics of the receptor. (e) Analysis of the leptin-docking residues on CRH2 domain, which has been shown to be critical in leptin binding and signal generation (8) suggest that our docking results agree to a certain extent with the experimental data on the effect of leptin on cells. Leptin docked predominantly to the residues in the N-terminal part of the CRH2 domain which has been reported to contain the leptin-binding site (8, 9, 46); the AA residue 567 (Asn), found to be involved in docking with leptin, has been reported in earlier mutation studies to be critical in signal transduction (47). Furthermore, earlier mutation studies have reported that the amino acid residues 35(Lys in human), 41 (Phe), 138 (Trp in human), 142/143 (leu/Ser in human) in leptin that were involved in docking with the receptor in our study, were critical in leptin-receptor binding and signaling (47, 48). Further, the amino acids identified to interact with CRP are present in the internal CRH2–FN III domain 2 which is reported to be critical in receptor dimerization and activation (8). Earlier cell-based studies had suggested that CRP attenuated leptin signaling (36). Since CRP is reported to bind to leptin, this effect could either be due to sequestration of leptin by CRP thereby reducing its availability, or due to a direct effect of CRP on the leptin receptor, a distinction which could not be resolved in an experimental system. The latter possibility is supported by our docking studies, which showed that mCRP docks to membrane proximal FN III domain, which is critical in receptor activation and signaling.

There is increasing evidence indicating dissociation of the pentameric CRP to the mCRP in tissues (49–51) including brain (52) and the role of mCRP in inflammation (4, 51, 53) and angiogenesis (54, 55). mCRP has been reported to bind to cells through cell surface molecules, such as CD16 (56) and α_V_β_3_ integrin (3). Presence of modified CRPs in plasma has been reported in obese human subjects (57). It, therefore, appears that the CRP, particularly the smaller mCRP, could attenuate leptin receptor activation independent of its ability to bind to leptin which might contribute to leptin resistance. Further experiments would be required to demonstrate its effect on leptin signaling.

Soluble leptin receptor to which CRP is found to bind is generated by shedding of the extracellular domain of membrane-anchored Ob R. Immunoblotting studies showed two bands of 121 and 136 kDa, corresponding to sOb R. These were also found in the CRP co-precipitate. Human sOb R has been reported to have a molecular weight of around 140 kDa. However, molecular weights ranging from 110 kDa to 290–300 kDa have also been reported (58–61). This difference has been attributed to differences in glycosylation of the core protein (61, 62). The data in this study are comparable to that reported earlier using a different antibody against sOb R (41). sOb R in plasma is considered as an important protein that binds to leptin and regulates its availability at target sites. But it is not clear how CRP-sOb R binding can influence the availability of sOb R for leptin sequestration.

The relevance of these binding studies was further examined by analyzing the levels of leptin, CRP, and sOb R in serum of non-obese and obese non-morbid human subjects. While a positive correlation between the levels of leptin and CRP was observed, there was a negative correlation between the levels of leptin and its soluble receptor. As reported in earlier studies (45), this appears to be related to obesity as indicated by a positive correlation between BMI and leptin and CRP. Although previous studies have demonstrated elevated CRP levels in obesity, the possibility of obesity associated co-morbidities contributing to this elevation could not be excluded. In the present study, the subjects did not have any co-morbidities, hence the increase in CRP levels is more likely to be linked to the expansion of adipose tissue. Unlike CRP, serum level of sOb R, is decreased in obese subjects compared to that of lean subjects. The levels of sOb R in plasma have been reported to be differentially regulated in various metabolic diseases (44). While our results on decrease in sOb R in obese subjects agree with those reported earlier (63), there are reports showing no change (64) and increased (65) levels of sOb R. These variations may be due to associated morbidity as evidenced by increase in plasma levels of sOb R in obese subjects with non-alcoholic steatosis (65). In the present study, obese subjects did not have any co-morbidity and any confounding effects were minimal. It has been shown that ADAM10/17 dependent cleavage of extracellular domain of the cell surface Ob R is the source of plasma sOb R (13). Change in ADAM 10 activity as in lipotoxicity or apoptotic conditions can contribute to changes in levels of sOb R (44). Changes in levels of cell surface Ob R is another factor determining the extent of its shedding and the serum level of sOb R is a reflection of its level of membrane expression (44). Thus the decrease in level of sOb R in obese subjects without any co-morbidity, observed in the present study, may reflect a lower level of membrane expression of Ob R. It is also possible that binding of CRP to membrane Ob R may protect against proteolytic cleavage resulting in lower levels of sOb R.

The negative correlation between BMI and sOb R suggests that the contribution of sOb R in the plasma in impeding the flow of leptin is relatively less compared to the effect of CRP which is elevated in obese subjects. In the light of these results on binding of CRP, particularly mCRP, to the cell surface domain of Ob R, it is possible that increased levels of CRP in obese subjects might contribute to diminished response to leptin in obese subjects.

The interaction of CRP with Ob R assumes particular significance in the context of the role of leptin in the interface of immune function, inflammation, and metabolism. Apart from its well-known effect as key regulator of metabolism, leptin, a pro-inflammatory cytokine and an activator of the immune system, is implicated in a number of inflammatory and immune disorders (66). Its levels are elevated in obese conditions and it mediates the pro-inflammatory state of obesity and associated pathophysiology. As leptin can stimulate CRP production by hepatocytes and the vasculature (24), these results suggest a possible regulatory system where binding of CRP to the leptin receptor may modulate the pleiotropic effects of leptin at multiple target sites. This may have implications in both adipose tissue physiology as well as pathogenesis of obesity related diseases.

## Ethics Statement

The study was approved by the Institutional Ethics Committee of the Sri Ramachandra University (Ref. IEC-NI/12/DEC/31/62) and all subjects provided informed written consent. The study was carried out in accordance with the ethical standards of the Institutional Ethics Committee and with the 1964 Helsinki declaration and its later amendments or comparable ethical standards.

## Author Contributions

MS, SS, and RR conceived and designed the study. Docking and binding studies and analysis by AS, AP, and MS. Sample collection and data analysis by MS, SS and RR. Manuscript written by MS, SS, and RR.

## Conflict of Interest Statement

The authors declare that the research was conducted in the absence of any commercial or financial relationships that could be construed as a potential conflict of interest.

## Supplementary Material

Figure S1 Immunoblot analysis of co-precipitated soluble leptin receptor (sOb) R C-reactive protein (CRP) was immunoprecipitated from human serum, subjected to 7.5% SDS-PAGE, followed by Western blotting, probed with anti-sOb R, and located by ECL **(B)** Negative controls: anti-CRP immunoprecipitate probed without anti-sOb R (only HRP conjugated secondary antibody) served as negative control **(C)** for identification of co-precipitated sOb R **(B)**. Anti-sOb R immunoprecipitate, probed without anti-CRP (only HRP conjugated secondary antibody) served as negative control **(A)** for identification of co-precipitated CRP (Figure 6D).
