# Supplementary figures and images for: C-Reactive Protein (CRP) and Leptin Receptor in Obesity: Binding of Monomeric CRP to Leptin Receptor
Source: Front Immunol. 2018 May 29;9:1167. doi: 10.3389/fimmu.2018.01167 (PMC5992430; doi:10.3389/fimmu.2018.01167)

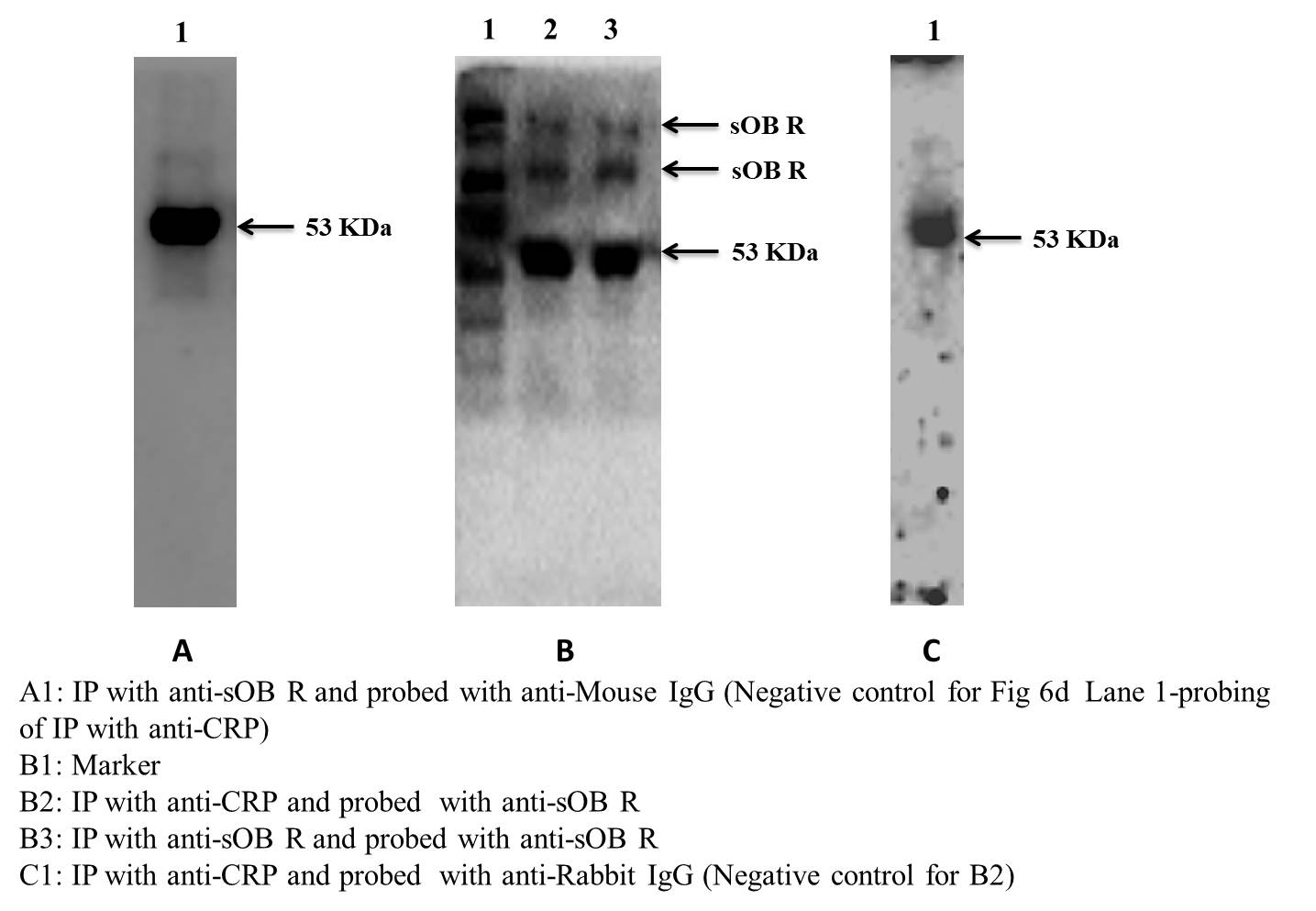

Supplement: Figure S1 — Immunoblot analysis of co-precipitated soluble leptin receptor (sOb) R C-reactive protein (CRP) was immunoprecipitated from human serum, subjected to 7.5% SDS-PAGE, followed by Western blotting, probed with anti-sOb R, and located by ECL (B) Negative controls: anti-CRP immunoprecipitate probed without anti-sOb R (only HRP conjugated secondary antibody) served as negative control (C) for identification of co-precipitated sOb R (B). Anti-sOb R immunoprecipitate, probed without anti-CRP (only HRP conjugated secondary antibody) served as negative control (A) for identification of co-precipitated CRP (Figure 6D). [file image_1.jpeg]
