# Supplementary material for: C-Reactive Protein (CRP) and Leptin Receptor in Obesity: Binding of Monomeric CRP to Leptin Receptor
Source: Front Immunol. 2018 May 29;9:1167. doi: 10.3389/fimmu.2018.01167 (PMC5992430; doi:10.3389/fimmu.2018.01167)
Supplement: Supplementary file 2 [file table_1.docx]

**S Table 1** . Amino acid residues involved in docking of leptin and C-reactive protein to leptin receptor. Amino acid residues involved in interactions between different domain structures of leptin receptor and each of the interacting protein and the non-bond interactions are given (a) Leptin receptor (CRH2) – CRP(monomer) (b) leptin receptor (CRH2)-leptin (c)leptin receptor (CRH2)-CRP (pentamer) (d) Leptin receptor (CRH2-FNIII)- CRP (monomer) ( e) Leptin/Leptin receptor (L-CRH2-FNIII)- CRP (monomer)

**S Table 1 (a)**

**Target – Leptin receptor (CRH2) (PDB ID – 3V6O A chain), Ligand – C- reactive protein single chain (PDB ID – 1GNH)**

***Z rank score -*115.571*; No. of non-bond interactions- 32***

| *Sl.No* | *Name* | *Distance* | *Category* |
| --- | --- | --- | --- |
| 1 | A:GLN571:HE21 - CRPA:GLN150:OE1 | 2.02481 | Hydrogen Bond |
| 2 | A:LYS614:HZ2 - CRPA:ASP140:OD1 | 2.44052 | Hydrogen Bond |
| 3 | A:LEU619:HN - CRPA:GLY79:O | 1.95202 | Hydrogen Bond |
| 4 | A:GLY620:HN - CRPA:ASP60:OD1 | 2.25795 | Hydrogen Bond |
| 5 | A:TRP622:HE1 - CRPA:GLU138:OE2 | 2.20265 | Hydrogen Bond |
| 6 | A:TRP625:HE1 - CRPA:GLU147:OE2 | 2.05348 | Hydrogen Bond |
| 7 | CRPA:LYS31:HZ3 - A:ASP475:OD1 | 2.65193 | Hydrogen Bond |
| 8 | CRPA:LYS57:HZ3 - A:ASN433:OD1 | 1.75185 | Hydrogen Bond |
| 9 | CRPA:GLN59:HE22 - A:SER533:O | 2.91486 | Hydrogen Bond |
| 10 | CRPA:LYS69:HN - A:GLU587:OE1 | 2.87976 | Hydrogen Bond |
| 11 | CRPA:THR76:HG1 - A:ASP617:O | 2.58001 | Hydrogen Bond |
| 12 | CRPA:SER80:HG - A:ASP617:OD2 | 2.30247 | Hydrogen Bond |
| 13 | A:SER533:HB2 - CRPA:GLN59:OE1 | 2.49182 | Hydrogen Bond |
| 14 | A:ARG612:HD1 - CRPA:GLU147:OE2 | 2.77571 | Hydrogen Bond |
| 15 | A:GLY620:HA2 - CRPA:ASP60:OD1 | 2.20651 | Hydrogen Bond |
| 16 | A:TYR621:HA - CRPA:ASP60:OD2 | 2.75741 | Hydrogen Bond |
| 17 | A:TRP622:HD1 - CRPA:ASP140:OD2 | 1.72456 | Hydrogen Bond |
| 18 | CRPA:PRO29:HD2 - A:ASP475:O | 2.95285 | Hydrogen Bond |
| 19 | CRPA:LYS31:HE2 - A:ASP475:OD1 | 2.83054 | Hydrogen Bond |
| 20 | CRPA:LYS57:HE1 - A:ILE434:O | 2.74587 | Hydrogen Bond |
| 21 | CRPA:THR76:HB - A:ASP617:O | 2.71956 | Hydrogen Bond |
| 22 | CRPA:SER141:HB2 - A:TYR621:OH | 2.73974 | Hydrogen Bond |
| 23 | A:ARG573:NH1 - CRPA:GLU147:OE2 | 4.95446 | Electrostatic |
| 24 | A:ARG612:NH1 - CRPA:ASP140:OD2 | 4.61472 | Electrostatic |
| 25 | A:ARG612:NH1 - CRPA:GLU147:OE2 | 2.52653 | Electrostatic |
| 26 | A:LYS614:NZ - CRPA:ASP60:OD2 | 4.21035 | Electrostatic |
| 27 | A:LYS614:NZ - CRPA:ASP140:OD2 | 5.31133 | Electrostatic |
| 28 | CRPA:LYS31:NZ - A:ASP475:OD2 | 4.12174 | Electrostatic |
| 29 | CRPA:ASP60:OD2 - A:TYR621 | 4.48078 | Electrostatic |
| 30 | CRPA:PRO29 - A:ILE476 | 4.90857 | Hydrophobic |
| 31 | CRPA:ARG47 - A:MET585 | 5.20759 | Hydrophobic |
| 32 | CRPA:ARG58 - A:VAL529 | 4.14968 | Hydrophobic |

A-Leptin Receptor (CRH2 domain) CRP-A-C-Reactive Protein (monomer A)

**S Table 1 (b)**

**Target – Leptin receptor (PDB ID – 3V6O A chain) , Ligand – Leptin (PDB ID – 1AX8) – blind docking**

***Z Rank score : -113.578 ;Non bond interactions : 20***

| *Sl.No* | *Name* | *Distance* | *category* |
| --- | --- | --- | --- |
|  | A:LYS614:HZ2 - L A:ASP135:OD2 | 2.63677 | Hydrogen Bond;Electrostatic |
|  | L A:LYS35:NZ - A:ASP590:OD2 | 5.32957 | Electrostatic |
|  | A:TYR589:HH - L A:PHE41:O | 2.36113 | Hydrogen Bond |
|  | A:LYS614:HZ3 - L A:ASP135:OD1 | 1.77081 | Hydrogen Bond |
|  | L A:TRP138:HE1 - A:GLY618:O | 2.77317 | Hydrogen Bond |
|  | A:ASN431:HA -L A:SER93:O | 1.87271 | Hydrogen Bond |
|  | A:SER435:HA - L A:LEU142:O | 1.82345 | Hydrogen Bond |
|  | A:SER435:HA - L A:SER143:O | 2.89349 | Hydrogen Bond |
|  | A:SER435:HB1 - L A:SER143:O | 2.49289 | Hydrogen Bond |
|  | L A:LYS33:HE1 - A:ASN567:OD1 | 2.02607 | Hydrogen Bond |
|  | L A:LYS94:HA - A:ASN431:O | 2.14901 | Hydrogen Bond |
|  | L A:SER143:HB1 - A:ASN433:OD1 | 2.15691 | Hydrogen Bond |
|  | L A:SER143:HB2 - A:ILE434:O | 1.99954 | Hydrogen Bond |
|  | A:TYR621 - L A:TRP138 | 5.51605 | Hydrophobic |
|  | A:PRO525 - L A:ILE3 | 4.3169 | Hydrophobic |
|  | A:TYR621 - L A:LEU49 | 5.09557 | Hydrophobic |
|  | A:TRP622 - L A:PRO43 | 5.05883 | Hydrophobic |
|  | L A:PHE41 - A:LEU616 | 3.44631 | Hydrophobic |
|  | L A:TRP138 - A:LEU619 | 4.6631 | Hydrophobic |
|  | L A:TRP138 - A:VAL534 | 5.29611 | Hydrophobic |

A –leptin receptor (CRH2), L-Leptin

**S Table1 (c)**

**Target – Leptin receptor (CRH2) (PDB ID – 3V6O A chain), Ligand – C- reactive protein (pentamer) (PDB ID – 1GNH)**

***Z rank score -*109.101*; No. of non-bond interactions- 13***

| *Sl.No* | *Name* | *Distance* | *Category* |
| --- | --- | --- | --- |
|  | LRA:LYS555:NZ - C:GLU88:OE2 | 5.59904 | Electrostatic |
|  | B:THR90:HG1 - LRA:ASN550:O | 2.75177 | Hydrogen Bond |
|  | C:GLU88:HN - LRA:ILE547:O | 2.64235 | Hydrogen Bond |
|  | LRA:THR548:HA - C:GLU88:OE1 | 2.49197 | Hydrogen Bond |
|  | LRA:ILE551:HA - B:THR90:OG1 | 1.61288 | Hydrogen Bond |
|  | LRA:PRO599:HD2 - B:PRO87:O | 2.28694 | Hydrogen Bond |
|  | LRA:PRO601:HA - B:SER45:O | 2.1781 | Hydrogen Bond |
|  | LRA:VAL633:HA - C:PRO87:O | 2.60221 | Hydrogen Bond |
|  | B:SER45:HB1 - LRA:VAL600:O | 2.74165 | Hydrogen Bond |
|  | LRA:PRO599 - B:VAL89 | 4.02061 | Hydrophobic |
|  | LRA:VAL633 - C:VAL89 | 4.15733 | Hydrophobic |
|  | LRB:VAL89 - A:LEU553 | 4.4566 | Hydrophobic |
|  | C:VAL86 - LRA:ILE549 | 4.55978 | Hydrophobic |

LR-Leptin Receptor (CRH2 domain ) , C,B,A –CRP(pentamer)

**S Table1 (d)**

**Target – Leptin receptor (Modelled(LR (CRH2-Fn) ) , Ligand – CRP (PDB ID – 1GNH)**

***Z rank score -*121.633*; No. of non bond interactions- 24***

| *Sl.No* | *Name* | *Distance* | *Category* |
| --- | --- | --- | --- |
|  | LR :LYS766:NZ - A:GLU81:OE2 | 4.20037 | Electrostatic |
|  | LR:LYS794:NZ - A:ASP60:OD2 | 4.21114 | Electrostatic |
|  | LR:LYS794:NZ - A:ASP140:OD2 | 3.24611 | Electrostatic |
|  | A:LYS31:NZ - LR:ASP475:OD2 | 5.49947 | Electrostatic |
|  | LR:ILE479:N - A:THR27:O | 2.59003 | Hydrogen Bond |
|  | A:LYS28:HZ1 - LR:SER478:OG | 2.03731 | Hydrogen Bond |
|  | LR:SER518:CB - A:GLN1:OE1 | 3.79421 | Hydrogen Bond |
|  | LR:PRO525:CA - A:LEU26:O | 3.2108 | Hydrogen Bond |
|  | LR:PRO526:CD - A:LEU26:O | 3.24106 | Hydrogen Bond |
|  | LR:LYS766:CE - A:GLU81:OE2 | 3.47876 | Hydrogen Bond |
|  | A:PRO25:HA - LR:ASP523:O | 3.08725 | Hydrogen Bond |
|  | A:LYS28:HE2 - LR:ILE479:O | 1.8735 | Hydrogen Bond |
|  | A:PRO29:HD2 - LR:PRO477:O | 1.40422 | Hydrogen Bond |
|  | A:THR46:HA - LR:ASP764:OD2 | 2.66463 | Hydrogen Bond |
|  | A:GLY48:HA1 - LR:TYR765:O | 2.7145 | Hydrogen Bond |
|  | A:ARG58:HA - LR:GLU805:O | 2.81632 | Hydrogen Bond |
|  | A:GLN59:HA - LR:ILE804:O | 2.06352 | Hydrogen Bond |
|  | A:LYS28:NZ - LR:HIS480 | 4.35886 | Electrostatic |
|  | A:LYS191:NZ - LR:HIS517 | 3.3392 | Electrostatic |
|  | A:ASP60:OD2 - LR:TYR807 | 4.2384 | Electrostatic |
|  | LR:LEU767:CD2 - A:PHE66 | 3.18264 | Hydrophobic |
|  | LR A:PRO25 - :LEU522 | 4.89665 | Hydrophobic |
|  | A:ARG58 - LR:PRO803 | 4.75252 | Hydrophobic |
|  | A:PHE66 - LR:LYS766 | 4.62767 | Hydrophobic |

LR -Leptin Receptor modelled(CRH2-FN III) , A –C-Reactive Protein (monomer A )

**S Table1 (e)**

**Target – Leptin – Leptin receptor (LR(CRH2-Fn III)) complex, Ligand – C- reactive protein single chain (PDB ID – 1GNH)**

***Z rank score -*129.234*; No. of non bond interactions- 26***

| *Sl.No* | Name | Distance | Category |
| --- | --- | --- | --- |
|  | L A:LYS35:HZ3 – CRP A:GLU197:OE2 | 2.23068 | Hydrogen Bond;  Electrostatic |
|  | LR:LYS766:NZ - CRP A:GLU147:OE2 | 3.64981 | Electrostatic |
|  | CRP A:LYS122:NZ - LR:ASP832:OD2 | 4.44225 | Electrostatic |
|  | LR:SER763:HG - CRP A:GLY79:O | 2.89303 | Hydrogen Bond |
|  | LR:LYS766:HZ1 - CRP A:GLU147:OE1 | 2.39009 | Hydrogen Bond |
|  | LR:GLN830:HE22 - CRP A:SER120:OG | 3.07589 | Hydrogen Bond |
|  | L A:LYS35:HZ2 - CRP A:GLU197:OE1 | 2.98256 | Hydrogen Bond |
|  | CRP A:LYS13:HZ1 - L A:LYS35:O | 1.83786 | Hydrogen Bond |
|  | CRP A:LYS122:HZ1 – LR :ASP832:O | 1.31773 | Hydrogen Bond |
|  | CRP A:ASN145:HD21 - L A:GLU122:OE1 | 2.65323 | Hydrogen Bond |
|  | LR:PRO762:HD1 - CRP A:ASP60:OD2 | 2.38808 | Hydrogen Bond |
|  | LR:LYS766:HE1 - CRP A:GLU147:OE2 | 1.94506 | Hydrogen Bond |
|  | LR:SER790:HA - CRP A:GLN59:OE1 | 2.98394 | Hydrogen Bond |
|  | L A:GLY118:HA1 - CRP A:ASP16:O | 2.79747 | Hydrogen Bond |
|  | L A:TYR119:HA - CRP A:PHE146:O | 2.52415 | Hydrogen Bond |
|  | CRP A:SER120:HA - L A:ASN145:OD1 | 2.61539 | Hydrogen Bond |
|  | CRP A:THR17:HA - L A:SER117:O | 2.55178 | Hydrogen Bond |
|  | CRP A:LEU121:HA - LR:THR829:O | 2.27644 | Hydrogen Bond |
|  | CRP A:SER141:HB2 - LR :TYR769:O | 2.92585 | Hydrogen Bond |
|  | LR:MET768:SD - CRP A:ASP140:O | 2.74046 | Other |
|  | CRP A:ASP16:OD2 - L A:TYR119 | 3.51632 | Electrostatic |
|  | LR:TYR765:HH - CRP A:PHE66 | 2.69971 | Hydrogen Bond |
|  | LR:TYR765 - CRP A:PHE66 | 5.34841 | Hydrophobic |
|  | L A:LYS35 - CRP A:LYS13 | 4.91349 | Hydrophobic |
|  | L A:VAL36 - CRP A:LYS13 | 5.3044 | Hydrophobic |
|  | CRP A:ARG58 - LR:ILE788 | 3.87915 | Hydrophobic |

LR- Leptin Receptor(CRH2-FNIII), L-Leptin, CRP- C-Reactive Protein (monomer A chain)
