# Supplementary material for: C-Reactive Protein (CRP) and Leptin Receptor in Obesity: Binding of Monomeric CRP to Leptin Receptor
Source: Front Immunol. 2018 May 29;9:1167. doi: 10.3389/fimmu.2018.01167 (PMC5992430; doi:10.3389/fimmu.2018.01167)
Supplement: Supplementary file 3 [file table_2.docx]

**S Table 2 Anthropometric and Biochemical Characteristics of the Subjects**

|  | Lean | | Obese | | P | |
| --- | --- | --- | --- | --- | --- | --- |
| N | 32 | | 42 | |  | |
| Sex(M/F) | 21/11 | | 28/14 | |  | |
| Age(yrs) | 30.56 ± 0.86 | | 32.95± 0.87 | | (n.s) | |
| BMI  ( kg/m^2^) | 21.71 (19.63- 22.22) | | 33.15( 31.69 -36.28) | | <0.0001 | |
| W(M/F)cm | 84 (76.5-86) | 83.09±1.52 | 109(106-115.4) | 108.4±1.86 | 0.0001 | 0.0001 |
| WHR  (M/F) | 0.88±0.0073 | 0.903±0.012 | 0.98±0.009 | 0.90 ±0.014 | <0.0001 | (n.s) |
| TC(mg/dl) | 166.5( 144-187.5) | | 179( 158-198) | | (n.s) | |
| LDLc (mg/dl) | 111(89.25-129.3) | | 128.5 (112.3-141) | | 0.02 | |
| HDL (mg/dl) | 45.34±1.506 | | 39.69±1 | | 0.003 | |
| TG (mg/dl) | 101(66.25-149) | | 125.5(97.75-177.3) | | 0.0142 | |
| FBS (mg/dl) | 91.5 (87.25-95.75) | | 95 (89.5-105) | | 0.047 | |

Values presented as Mean± S.E.M or Median(IQR); p<0.05 considered significant( by unpaired “t”test or Mann Whitney-U test.
