# Supplementary material for: C-Reactive Protein (CRP) and Leptin Receptor in Obesity: Binding of Monomeric CRP to Leptin Receptor
Source: Front Immunol. 2018 May 29;9:1167. doi: 10.3389/fimmu.2018.01167 (PMC5992430; doi:10.3389/fimmu.2018.01167)
Supplement: Supplementary file 4 [file table_3.docx]

|  | Male | |  | Female | |  |
| --- | --- | --- | --- | --- | --- | --- |
|  | BMI<23 | BMI>25 | p | BMI<23 | BMI>25 | p |
| N | 20 | 28 |  | 11 | 14 |  |
| Leptin (ng/ml) | 11.45  (6.0-21.2) | 50.6  (32.3-59.9) | <0.0001 | 19.8  (16.8-21.8) | 61.5  (58.3-65.5) | <0.0001 |
| sOB R (ng/ml) | 27  (24.9-42.0) | 21.13  (16.02-27.7) | <0.005 | 19.5  (17.2-31) | 23.6  (14.3-28) | n.s |
| CRP (mg/dl) | 0.15  (0.11-0.23) | 0.55  (0.39-0.73) | <0.0001 | 0.185  (0.135-0.26) | 0.94  (0.62-1.67) | <0.0001 |

**S Table 3. Serum levels of Leptin, soluble Leptin Receptor (sOb R) and C-Reactive Protein(CRP) in male and female subjects.**

Values expressed as median (inter-quartile range). Comparison was done by the Mann-Whitney-U test.
